# Utilizing artificial intelligence in academic writing: an in-depth evaluation of a scientific review on fertility preservation written by ChatGPT-4

**DOI:** 10.1007/s10815-024-03089-7

**Published:** 2024-04-15

**Authors:** Myriam Safrai, Kyle E. Orwig

**Affiliations:** 1 Department of Obstetrics, Gynecology and Reproductive Sciences, Magee-Womens Research Institute, University of Pittsburgh School of Medicine, Pittsburgh, PA 15213 USA; 2 Department of Obstetrics and Gynecology, Chaim Sheba Medical Center (Tel Hashomer), Sackler Faculty of Medicine, Tel Aviv University, 52621 Tel Aviv, Israel

**Keywords:** Artificial intelligence (AI), Natural language processing, Fertility, Academic writing, ChatGPT

## Abstract

**Purpose:**

To evaluate the ability of ChatGPT-4 to generate a biomedical review article on fertility preservation.

**Methods:**

ChatGPT-4 was prompted to create an outline for a review on fertility preservation in men and prepubertal boys. The outline provided by ChatGPT-4 was subsequently used to prompt ChatGPT-4 to write the different parts of the review and provide five references for each section. The different parts of the article and the references provided were combined to create a single scientific review that was evaluated by the authors, who are experts in fertility preservation. The experts assessed the article and the references for accuracy and checked for plagiarism using online tools. In addition, both experts independently scored the relevance, depth, and currentness of the ChatGPT-4’s article using a scoring matrix ranging from 0 to 5 where higher scores indicate higher quality.

**Results:**

ChatGPT-4 successfully generated a relevant scientific article with references. Among 27 statements needing citations, four were inaccurate. Of 25 references, 36% were accurate, 48% had correct titles but other errors, and 16% were completely fabricated. Plagiarism was minimal (mean = 3%). Experts rated the article’s relevance highly (5/5) but gave lower scores for depth (2–3/5) and currentness (3/5).

**Conclusion:**

ChatGPT-4 can produce a scientific review on fertility preservation with minimal plagiarism. While precise in content, it showed factual and contextual inaccuracies and inconsistent reference reliability. These issues limit ChatGPT-4 as a sole tool for scientific writing but suggest its potential as an aid in the writing process.

**Supplementary Information:**

The online version contains supplementary material available at 10.1007/s10815-024-03089-7.

## Introduction

Millions of people now use artificial intelligence (AI)–based language models, such as ChatGPT, capable of mimicking human communication and generating coherent texts in seconds [1]. In response to specific prompts, ChatGPT can produce practically any kind of text, including scientific papers. As a result, ChatGPT could serve as a valuable tool for scientific writing tasks such as draft automation, article summarization, and translation, easing the writing activity of physicians and scientists. Nevertheless, implementing ChatGPT in scientific writing is a subject of debate in academia. For example, paper authorship has become controversial after ChatGPT was listed as an author in several research papers [2].

As a result, various studies have investigated the use of ChatGPT in academia. For instance, several studies have analyzed the possibility of detecting abstracts generated by ChatGPT-3.5 [3, 4], reporting a detection rate of ~ 70%. In addition, a study showed ChatGPT-3’s content is not detected as plagiarism [5], which caused concern in the education field [6]. Research points to the limitations of using ChatGPT-3.5 for conducting systematic reviews due to its lack of understanding of the process and inability to look through the literature [7]. Additionally, ChatGPT-3.5 demonstrated poor competence in providing accurate references on rheumatologic topics [8]. In March 2023, ChatGPT-4 was launched, allowing the process a greater word limit, a stronger ability to solve complex problems, and image recognition [9], in anticipation that it will be able to handle much more nuanced instructions and tackle some of the limitations of the previous versions.

Despite the wide use of ChatGPT, the literature on the scientific accuracy of different versions of ChatGPT writing in scientific/medical reviews is scarce. This is concerning as factual inaccuracies, ethical issues, and the spread of misinformation in medicine can impact the scientific knowledge base and patient care. Therefore, we aim to examine the accuracy of the scientific and medical data generated by ChatGPT-4 and the ability to write a scientific review on the topic of fertility preservation in response to prompts. We evaluated the accuracy of the references provided, plagiarism, the relevance of the topics, and the depth and currentness of the text produced by ChatGPT-4.

## Methods

Fertility preservation (FP) in men and prepubertal boys was chosen for two key reasons. First, fertility preservation for men is a well-known subject allowing the possibility for assessment of well-documented scientific facts. On the other hand, FP for prepubertal boys is a constantly evolving field with various knowledge gaps presenting an opportunity to analyze and interpret up-to-date research data. Second, as experts in FP, we can critically examine the validity and relevance of associated inquiries. This study is exempt from Institutional Review Board (IRB) review as it did not include any interaction or intervention with human subjects or access to identifiable private information.

### ChatGPT-4 article creation

**Fig. 1:**
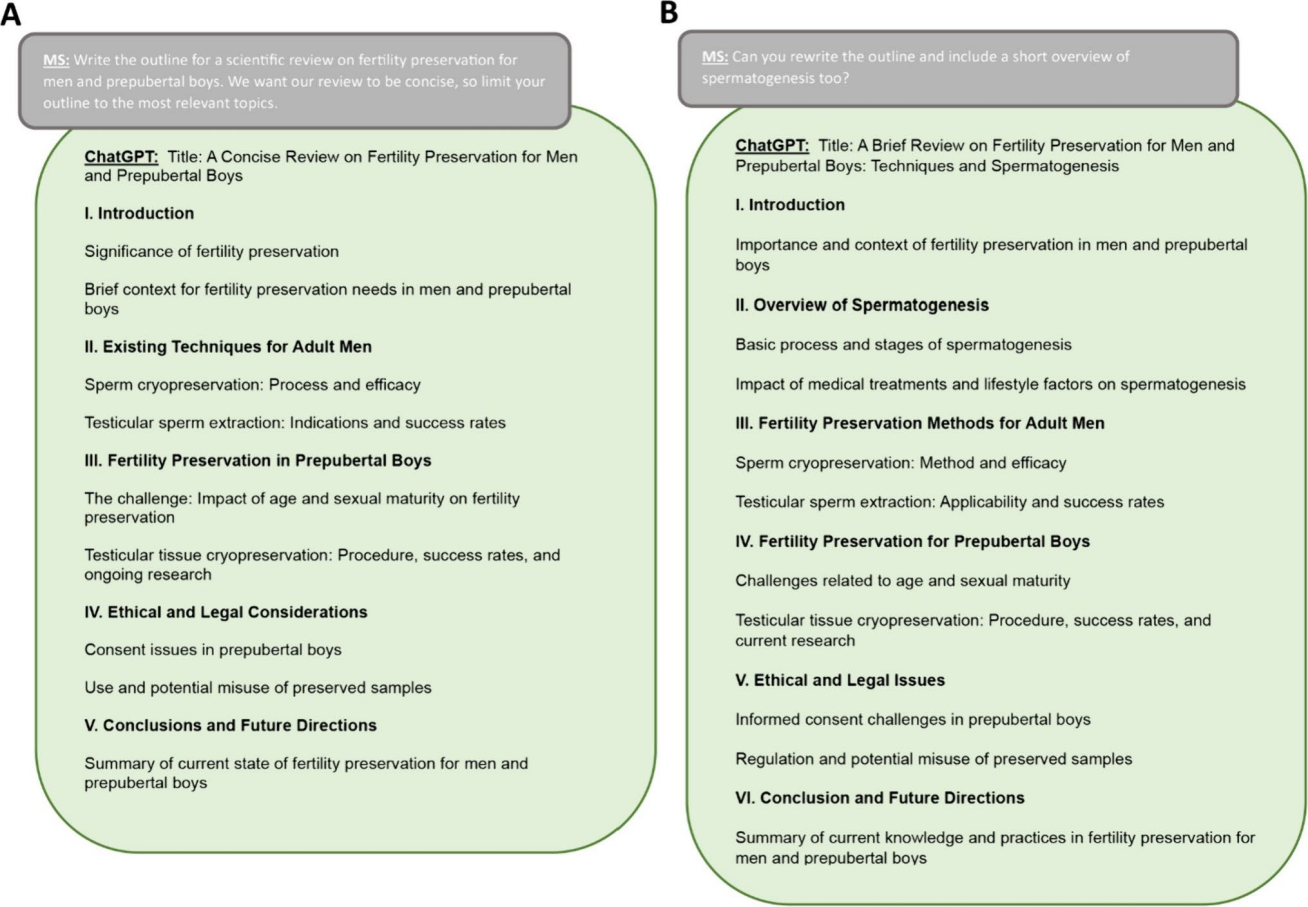
Response by ChatGPT-4 following a prompt to provide an outline for an article on fertility preservation for men and prepubertal boys. **A** First response to the prompt: “Write the outline for a scientific review on fertility preservation for men and prepubertal boys. We want our review to be concise, so limit your outline to the most relevant topics.” **B** Second response of ChatGPT following the specification to include spermatogenesis in the outline

**Fig. 2:**
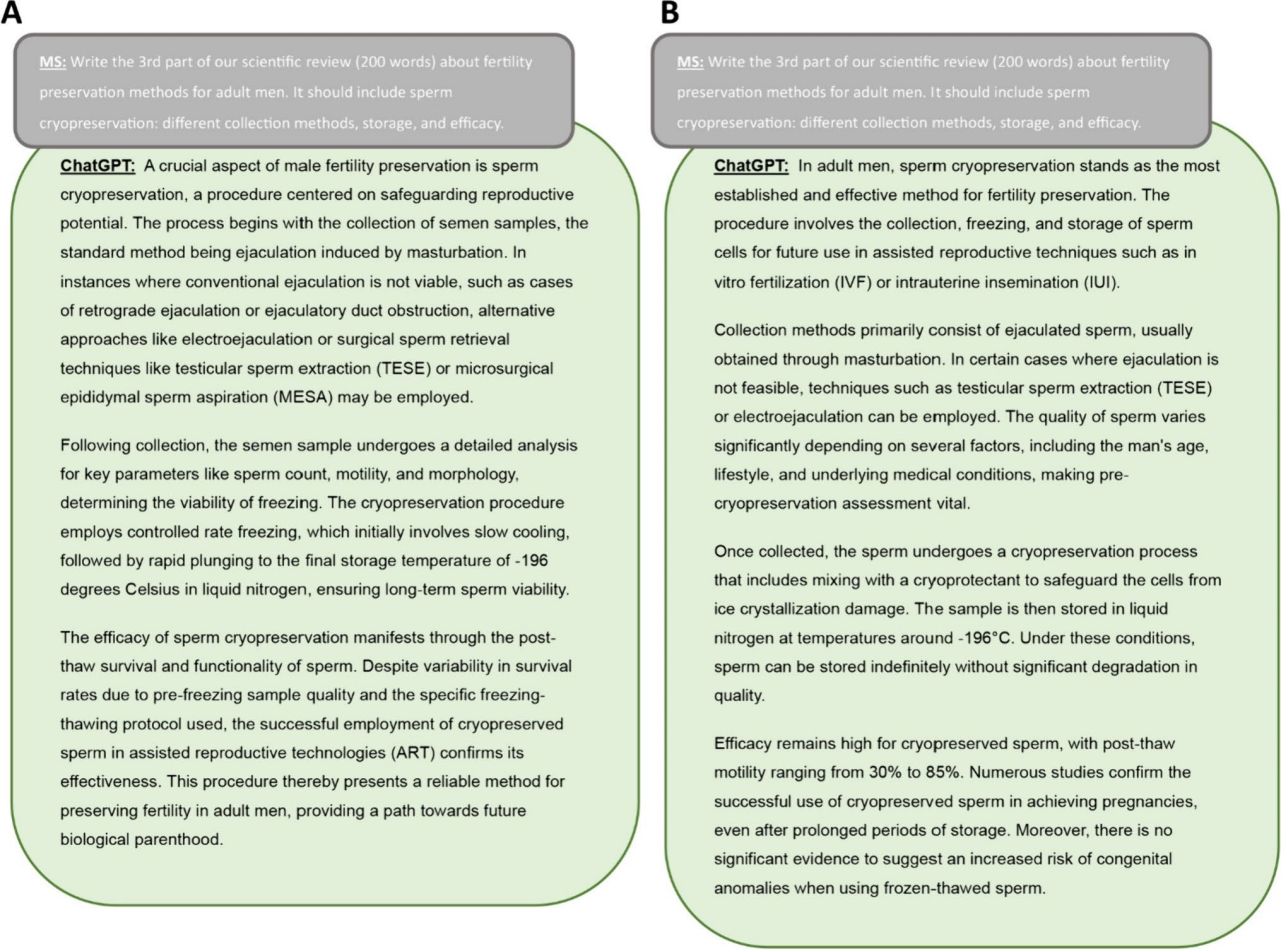
Various responses by ChatGPT to the same prompt on two different occasions

A scientific review on fertility preservation for men and prepubertal boys was created from ChatGPT-4 answers to prompts. All the prompts were written by a single author (MS) and detailed in Supplementary Information. First, ChatGPT-4 was asked to suggest an outline for this article using the following prompt: “Write the outline for a scientific review on fertility preservation for men and prepubertal boys. We want our review to be concise, so limit your outline to the most relevant topics.” Since the first outline did not include spermatogenesis, a key topic in male FP, we prompted ChatGPT to add it in a second outline (Fig. 1). The final article included 6 Sects. (1. Introduction, 2. Overview of Spermatogenesis, 3. Fertility Preservation Methods for Adult Men, 4. Fertility Preservation for Prepubertal Boys, 5. Ethical and Legal Issues, and 6. Conclusion), as suggested in the second outline generated by ChatGPT-4. The title of the outline and the subtitles generated by ChatGPT were used to build the different prompts used to trigger ChatGPT-4 to write each section separately. For example, the 3rd section, “Fertility Preservation Methods for Adult Men,” was generated in response to the prompt: “Write the 3rd part of our scientific review (200 words) about fertility preservation methods for adult men. It should include sperm cryopreservation: different collection methods, storage, and efficacy.” Following each answer, ChatGPT was prompted to provide 5 references for the section written, by various prompts such as “Give me 5 references for the last paragraph.” To assess the reproducibility of the answer of ChatGPT-4, it was prompted a second time with the same prompt provided earlier, and the first answer was the one included in the final article (Fig. 2).

### Assessment of the ChatGPT-4 article

#### Accuracy of the scientific and medical data

The article was reviewed by two experts in FP (MS and KEO). All the statements made by Chat-GPT-4 containing information that authors would typically support with a reference citation were selected and assessed for accuracy. The authors searched PubMed and Google Scholar to identify references to support those statements generated by ChatGPT-4. The evaluation was done by entering the phrase or section of the phrase such as important words from the paragraph into the search domain, and the results are presented in Supplementary Table 1.

#### Validation of ChatGPT-provided references

ChatGPT-4 was prompted to provide five references for each section. These were authenticated in PubMed or Google Scholar by the authors. When ChatGPT-4 provided the article’s title and additional parameters such as the journal name, author names, and DOI, all the different parameters were verified.

#### Plagiarism assessment

The manuscript generated by ChatGPT-4 was uploaded to two different online plagiarism checkers: “Grammarly” [10] and “Quetext” [11]. The overall percentage of plagiarism reported by each one was recorded. In addition, every sentence suggested as a potential for plagiarism was recorded as well as the reference that it may have plagiarized (Table 1).

**Table 1:** Plagiarism checker reports

Plagiarism checker	% of plagiarism	Possible plagiarized sentences	Reference title
Grammarly	4	“They have not yet undergone the process of”	Surface Sensing and Settlement Strategies of Marine Biofouling Organisms [35]
		“By delving into these topics, we hope to”	About the Center for Christianity in Business https://hc.edu/center-for-christianity-in-business/about-the-ccb/
		“From type A to type B spermatogonia, and”	Quantitative (stereological) study of incomplete spermatogenic suppression induced by testosterone undecanoate injection in rats [36]
		“This procedure involves the surgical removal of a small”	How neutologist treat Parkinson’s disease? https://drjeffsteinberg.com/how-neurologist-treat-parkinsons-disease/
Quetext	2	“The process begins with the collection of semen samples, the standard method being ejaculation induced by masturbation.”	The utilization of biological processes on farms to produce food and other products https://sciwheel.com/work/item/14940959/resources/17164735/pdf

#### Relevance, depth, and currentness of ChatGPT-4 writing on the main topics

The authors (MS and KEO) independently scored the relevance, depth, and currentness of the writing of ChatGPT-4 on a scale of 1–5, with 5 being the highest score. All the parameters were subjective but based on certain definitions. Relevance refers to the connection with the overall topic of the review and significance to the context of the paragraph where it appears. Depth of the article refers to the extent to which it provides comprehensive, detailed, and thorough information on the subject. Currentness is an evaluation of whether the information provided by ChatGPT-4 was up-to-date.

#### Data analysis

All collected data were entered and managed in Microsoft Excel (Version 2305, Microsoft Corp., Redmond, WA). Descriptive statistics, including percentages, were computed using built-in functions of Microsoft Excel.

## Results

ChatGPT-4 answered all the prompts within seconds of the request, generating relevant content and creating the following article:

***Title:*** *From Spermatogenesis to Cryopreservation: An Overview of Male Fertility Preservation Strategies and Anticipated Developments*

***Keywords: ****ChatGPT, AI Scientific Writing, AI in Reproductive Health, Male Fertility, Prepubertal Fertility*

I. *Introduction.*
  *Fertility preservation represents a critical concern within the broader framework of reproductive health, especially for those facing potential fertility risks. As advances in medicine continue to improve survival rates for conditions like cancer, the focus is increasingly shifting towards enhancing the quality of life post-recovery, of which preserving the ability to have biological children plays a significant part. In this context, the necessity for effective fertility preservation strategies for men and prepubertal boys cannot be overstated. The latter group particularly presents unique challenges as they have not yet undergone the process of spermatogenesis, limiting the application of techniques readily available for adults.*
  *This review aims to explore the latest scientific understanding, techniques, and ongoing research in fertility preservation for men and prepubertal boys. By delving into these topics, we hope to provide a comprehensive overview and highlight future directions in this pivotal area of reproductive medicine, where every advancement has the potential to drastically impact the lives of those grappling with fertility-threatening conditions.*
II. *Overview of Spermatogenesis.*
  *Fertility preservation critically hinges on a detailed understanding of spermatogenesis, the complex biological process underlying male fertility. This transformation unfolds within the seminiferous tubules of the testes, transitioning spermatogonial stem cells into mature spermatozoa.*
  *The initial proliferative phase involves spermatogonial stem cells undergoing self-renewing mitotic divisions and sequential differentiation, transitioning from type A to type B spermatogonia, and further into primary spermatocytes. In the subsequent meiotic phase, each primary spermatocyte undergoes two sequential meiotic divisions. Meiosis I results in a reduction of the diploid number of chromosomes to haploid, while Meiosis II segregates the sister chromatids into separate cells, generating four haploid secondary spermatocytes. Finally, the spermiogenic phase is marked by round spermatids undergoing critical morphological transformations to form mature spermatozoa. These changes encompass acrosome and flagellum development, nuclear condensation, and cytoplasmic shedding. Each stage of spermatogenesis, being sensitive to different factors, demands precision in fertility preservation strategies.*
III. *Fertility Preservation Methods for Adult Men.*
  *A crucial aspect of male fertility preservation is sperm cryopreservation, a procedure centered on safeguarding reproductive potential. The process begins with the collection of semen samples, the standard method being ejaculation induced by masturbation. In instances where conventional ejaculation is not viable, such as cases of retrograde ejaculation or ejaculatory duct obstruction, alternative approaches like electroejaculation or surgical sperm retrieval techniques like testicular sperm extraction (TESE) or microsurgical epididymal sperm aspiration (MESA) may be employed.*
  *Following collection, the semen sample undergoes a detailed analysis for key parameters like sperm count, motility, and morphology, determining the viability of freezing. The cryopreservation procedure employs controlled rate freezing, which initially involves slow cooling, followed by rapid plunging to the final storage temperature of -196 degrees Celsius in liquid nitrogen, ensuring long-term sperm viability.*
  *The efficacy of sperm cryopreservation manifests through the post-thaw survival and functionality of sperm. Despite variability in survival rates due to pre-freezing sample quality and the specific freezing–thawing protocol used, the successful employment of cryopreserved sperm in assisted reproductive technologies (ART) confirms its effectiveness. This procedure thereby presents a reliable method for preserving fertility in adult men, providing a path towards future biological parenthood.*
IV. *Fertility Preservation for Prepubertal Boys.*
  *In the context of fertility preservation, prepubertal boys present unique challenges. Principally, these pertain to their sexual immaturity and the associated absence of mature sperm. The leading method considered for fertility preservation in this demographic is testicular tissue cryopreservation. This procedure involves the surgical removal of a small portion of testicular tissue, which harbors spermatogonial stem cells (SSCs), and its subsequent freezing. These SSCs hold potential to initiate spermatogenesis in the future, when reimplanted or matured *in vitro*. Current research is exploring several promising options for utilizing cryopreserved testicular tissue. These include autologous transplantation, heterologous transplantation, and *in vitro* maturation (IVM) of testicular tissue or isolated SSCs.*
  *However, it is essential to underscore that these potential methods, although promising, are still primarily within the research domain. Several critical issues, including the risk of reintroducing malignancy and optimizing conditions for *in vitro* maturation, require further exploration and resolution. Nevertheless, the continuous advancement in this area fosters hope for more reliable and efficient fertility preservation options for prepubertal boys in the future.*
V. *Ethical and Legal Issues.*
  *Navigating the ethical and legal landscape of fertility preservation, especially in prepubertal boys, poses unique challenges. Informed consent is a complex issue, as the implications, potential benefits, and risks of an experimental technique like testicular tissue cryopreservation must be thoroughly understood by parents or guardians. Moreover, strict regulations are required to guard against potential misuse of cryopreserved samples. These include unambiguous rules regarding the authorization of sample use, sample transfer between institutions, international transport, and disposal of unused samples, ensuring the protection of individual rights and legal integrity.*
VI. *Conclusion.*
  *In conclusion, the field of fertility preservation continues to evolve, addressing unique challenges associated with adult men and prepubertal boys. Current practices like sperm cryopreservation and testicular sperm extraction have proven valuable for adults, while methods such as testicular tissue cryopreservation are being explored for prepubertal boys. The ongoing research and anticipated advancements in this field reflect a promising trajectory, as the primary aim remains to ensure viable options for all individuals faced with potential fertility threats, thereby enhancing their quality of life.*

### Scientific and medical accuracy

#### Title and keywords

**Fig. 3:**
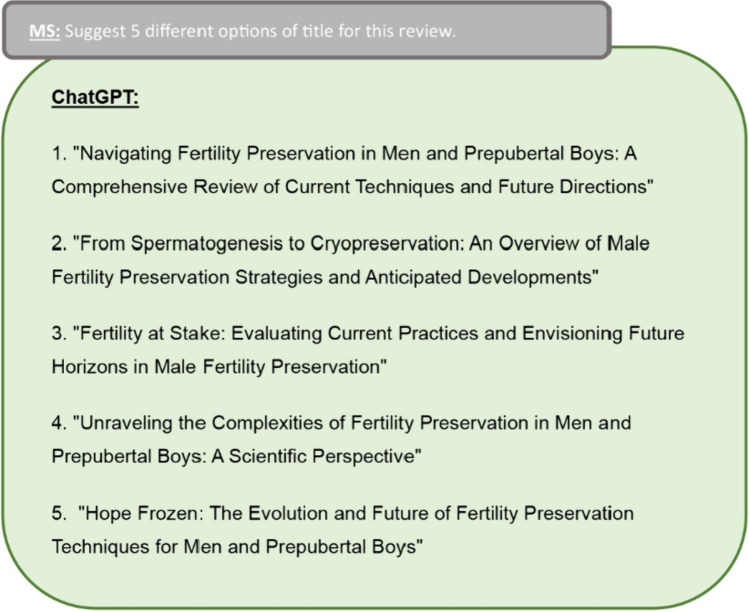
Response by ChatGPT-4 to a request for five potential titles for the review

ChatGPT-4 was prompted to suggest 5 potential titles for this article (Fig. 3). They were all accurate for the topic of this article, although the reviewers/authors may have phrased some of the titles differently. The keywords suggested by ChatGPT: “ChatGPT, AI Scientific Writing, AI in Reproductive Health, Male Fertility, Prepubertal Fertility” were all found relevant to the article topics by both authors.

#### Main article

ChatGPT does not provide source references when writing a scientific review. The authors identified a total of 27 statements that they believed required citations (Supplementary Table 1). Four out of 27 statements (15%) were incorrect. The first mistake was made in describing the second meiosis of the spermatogenesis process “…Meiosis II segregates the sister chromatids into separate cells, generating four haploid secondary spermatocytes.” The claim should be that the second meiosis results in four spermatids [12]. Following this paragraph when ending the “Overview of Spermatogenesis,” ChatGPT-4 summarized: “Each stage of spermatogenesis, being sensitive to different factors, demands precision in fertility preservation strategies.” This statement is inaccurate—while spermatogenesis is indeed a multi-step process, fertility preservation is achieved by sperm cryopreservation or in the research phase for prepubertal patients by cryopreservation of testicular tissues, which contain spermatogonial stem cells. There are currently no clinical options to preserve and restore fertility from other stages of spermatogenesis. In the third section on fertility preservation methods for adults, ChatGPT-4 describes other options to procure sperm in case of retrograde ejaculation or ejaculatory duct obstruction and listing “electroejaculation or surgical sperm retrieval techniques like testicular sperm extraction (TESE) or microsurgical epididymal sperm aspiration (MESA).” Electroejaculation is not the treatment for retrograde ejaculation or ejaculatory duct obstruction [13, 14]. When describing the semen assessment, ChatGPT-4 wrote: “Following collection, the semen sample undergoes a detailed analysis for key parameters like sperm count, motility, and morphology, determining the viability of freezing.” Those analyses do not evaluate the viability of freezing but the quality of the sample pre-freezing or post-thaw. All the other statements were validated by the authors, and references were provided when appropriate. For detailed information, see Supplementary Table 1.

It is noteworthy that when asked the same question twice, ChatGPT responded differently each time (Fig. 2). If the response to a question is not acceptable to the author, there is an option to reload the question and receive another response. This is an interesting and potentially valuable feature but indicates that ChatGPT would write two completely different articles if given the same series of instructions at two different times.

#### Reference validation

ChatGPT-4 generated 5 references for each section, resulting in 25 references distributed across the five sections of the article. Each reference incorporates the article title, publication journal, volume, pages, and author information. Furthermore, 20 out of the 25 references also included a DOI. Nine of 25 references (36%) were existing references, and all their parameters were correct. In contrast, 4 (16%) were fictitious references invented by ChatGPT-4. One example is the reference suggested for the introduction section: “Shetty, G., Meistrich, M. L., & Lipshultz, L. I. Hormone-based fertility preservation for boys: the role of the testis and potential alternatives, Pediatric Blood & Cancer, 55(3), 399–401, https://doi.org/10.1002/pbc.22492.” Despite the title initially appearing relevant to the subject matter and the authors being researchers who work in this field, no article with such a title exists. That could be misleading even to a researcher with expertise in the field. Furthermore, the provided DOI corresponds to a completely different article on fertility preservation with different authors and a different title than the one provided.

The remaining 12 references had a correct title but other fictitious parameters, such as the reference: “Shenfield, F., Pennings, G., Cohen, J., Devroey, P., de Wert, G., Tarlatzis, B. ESHRE's good practice guide for cross-border reproductive care for centers and practitioners, Human Reproduction, 27(7), 3101–3104, 10.1093/humrep/des114” in the fifth section discussing ethical and legal issues. The title and the journal provided are correct, as are the two first authors listed by ChatGPT. However, while the other authors (Cohen, J., Devroey, P., de Wert, G., and Tarlatzis, B.) have indeed written relevant papers in the field and even other ESHRE guidelines, they are not the co-authors of this specific article. In total, the majority of the references (21/25, 84%) had a title that could be found in PubMed or Google Scholar, but only 36% had completely accurate reference information.

#### Plagiarism detection

Both online plagiarism detection tools reported a low percentage of plagiarism (4% and 2% for Grammarly and Qutext, respectively) (Table 1). Grammarly [10] listed 4 matches, while Quetex [11] listed only one. The four sentences detected as potential plagiarism by Grammarly were short, with a mean of 8.25 words, and included general content only, as can be seen in Table 1. On the other hand, Qutext listed one longer sentence: “The process begins with the collection of semen samples, the standard method being ejaculation induced by masturbation.” However, as the content reported by Grammarly, Qutext’s content too was common knowledge, therefore not taking the work of another and not accounted as actual plagiarism [15, 16].

#### Subjective scoring of the authors

Both authors, experts in fertility preservation, gave the highest score of 5 for the relevance of the topics raised in the article and noted that the subjects appear in other peer-reviewed articles on those subjects [17–19]. Both authors attributed the same score of 3 to the currentness (i.e., up to date) of data provided by ChatGPT-4. They explained that the report on sperm cryopreservation did not include recent methods, such as vitrification, used in some laboratories today. One of the authors scored the depth of the manuscript provided with a 2 and the second with a 3. Both authors reported that the various adapted subjects mentioned in the outline were subsequently explained in a shallow manner without showing a deep understanding of the subjects.

## Discussion

This study demonstrated that ChatGPT-4 could generate accurate statements (85%) in the field of FP, outlining relevant topics and elaborating on each of them accordingly. This finding aligns with previous studies reporting ChatGPT’s ability to pass different medical exams [20, 21], scoring 60–92% of correct answers and providing appropriate medical recommendations in ~ 80% of the cases [22, 23]. Specifically in obstetrics and gynecology topics, ChatGPT can help users access clinically related information [24], achieving a high score with 94% of correct answers to frequently asked questions on infertility topics [25]. However, despite the high rate of accurate data and the relevance of the topics raised, the remaining inaccuracies make ChatGPT-4 inappropriate to be used as a stand-alone tool to generate reliable scientific reviews. Moreover, some aspects in each response to the same prompt were not covered in the other response, suggesting that either response may be incomplete. In addition, both reviewers of the text generated by ChatGTP-4 found it shallow and not up to date, which could impact patient care if used in clinical practice. A lag in the learning phase for AI with the current model must be weighed against the rapid new research that changes practice standards. This all implies that while ChatGPT-4 may be used to suggest the relevant subject for a scientific article, a careful review of the writing by an expert in the field is required to avoid misleading information and provide synthesis of ideas and depth of discussion.

Of the 25 references provided by ChatGPT-4, only 9 were completely accurate. Four were completely fictitious and would probably be discovered by a reasonably competent researcher in the field. However, it is concerning that 12 of 25 references were partially correct and might reasonably be missed by someone with expertise in the field who recognizes the author names together with a title that is relevant to the field. This deficiency was also highlighted in previous assessments of ChatGPT writing [26, 27]. In the world of AI, those are defined as hallucinations—a confident response by artificial intelligence that does not seem to be justified by its training data [28]. Inventing non-existing references is a critical flaw because they could be misleading to an uninformed author or even a reasonably informed author. Hallucinations are a well-known flaw of ChatGPT and other large language models (LLMs) and can be caused by several reasons. For example, it can happen when the LLM was trained on outdated or low-quality data, resulting in an inaccurate response, or equally if the prompt uses idioms or slang expressions that the model has yet to be trained on. However, the hallucinations pose an issue to the future use of Chat-GPT, as the falsified sources are not exposed unless Chat-GPT is asked about them directly. As there is no way to completely avoid hallucinations, it is recommended to verify every output to avoid inclusion of false information [29].

The possible plagiarism detection rate raised by two online detectors for our article was less than 5%. In addition, the assessment of the text suggested as plagiarism did not match the definition of plagiarism [15]. This part of our study corroborates the results of previous studies, where the text generated by ChatGPT-4 was not flagged as plagiarism [16, 30, 31]. Those results raised the concern that new plagiarism classifications may be needed, as rather than simply determining if a text is plagiarized, it may need to be assessed if it was generated by AI tools [32]. Consequently, different tools are currently being developed to detect texts generated by AI Chatbots [4, 33].

This study has several limitations. First, the subject chosen was fertility preservation, and the text was generated in response to specific prompts. However, different topics and other prompts are likely to result in different answers. Nevertheless, many of our observations were consistent with observations in other studies of ChatGPT performance on different subjects. Second, selecting the statement requiring references was subjective and thus susceptible to bias and variations. Third, requesting 5 references for each section may have forced ChatGPT to invent references if it could not find five relevant references in the data it was trained on [29]. Another potential limitation of the study is the authors’ subjective scoring of quality, relevance, and depth of ChatGPT-4 writing. Even though the authors are experts in the field, it is possible that other researchers in fertility might have made different selections or given a different grading. Despite these limitations, these findings represent an important step in assessing the benefits and limitations of using ChatGPT-4 when writing a scientific article on fertility preservation. It is recommended that future studies use different subjects, prompts, and experts in those areas to assess ChatGPT’s accuracy, further benefits, and limitations in scientific writing.

In conclusion, our finding shows the technical capability of ChatGPT-4 to rapidly produce texts for a scientific review on fertility preservation without being flagged as plagiarism. However, inaccurate statements and unpredictable errors in generating relevant citations raise serious concerns. As a result, the human author must be well-versed in the field to assess ChatGPT-4-generated responses to avoid misleading information. Each reference needs to be carefully validated for all its parameters to prevent the incorporation of hallucinations. Consequently, at this time, this technology cannot replace human scientific expertise. However, in other fields, it has already been demonstrated that it can shorten the time of different tasks, for example, by reducing the coding time from weeks to days [34]. Similarly, it has the potential to facilitate and accelerate the writing process by automating the task and producing a page of text within seconds if used judiciously. This raises the need for individuals and scientific communities to adapt and benefit from this new technology while avoiding adverse implications. We encourage others to attempt similar exploration and testing of this new AI tool on different subjects to understand their current limitations and capacity.

### Supplementary Information

Below is the link to the electronic supplementary material.

Supplementary file1 (DOCX 55 KB)

## Data Availability

The data underlying this article are available in the article and in its online supplementary material.
