# Supplementary material for: Utilizing artificial intelligence in academic writing: an in-depth evaluation of a scientific review on fertility preservation written by ChatGPT-4
Source: J Assist Reprod Genet. 2024 Apr 15;41(7):1871–80. doi: 10.1007/s10815-024-03089-7 (PMC11263262; doi:10.1007/s10815-024-03089-7)
Supplement: Supplementary file 1 — Supplementary file1 (DOCX 55 KB) [file 10815_2024_3089_MOESM1_ESM.docx]

**Supplementary Information.**

**List of prompts submitted to ChatGTP-4 to create the scientific article**

1. Write the outline for a scientific review on fertility preservation for men and prepubertal boys. We want our review to be concise, so limit your outline to the most relevant topics.

2. Can you rewrite the outline and include a short overview of spermatogenesis too?

3. Write the introduction for this concise review. Make it interesting and engage people in this topic. This is intended to be read by a scientific expert population in the field. It should include the importance and context of fertility preservation in men and prepubertal boys.

4. Great, can you give me 5 references for this section?

5. Please write the second section (200 words) of our scientific review about the overview of spermatogenesis. It should include: The process and stages of spermatogenesis. Start with a phrase explaining that it is important to understand spermatogenesis in the field of fertility preservation. Don't summarize at the end. Go deeper in the explanation as the readers of this review are fertility experts.

6. This is a very good response. Delete the last paragraph as it it redundant with the first phrase.

7. Perfect, give me 5 references for this section.

8. Write the 3rd part of our scientific review (200 words) about fertility preservation methods for adult men. It should include sperm cryopreservation: different collection methods, storage, and efficacy.

9. Great, provided me 5 references for this section.

10. Write the 4th part of our scientific review (200 words) about fertility preservation for prepubertal boys. It should include the challenges related to age and sexual maturity, the testicular tissue cryopreservation procedure and current research for future uses of the tissue.

11. Give me 5 references for the last paragraph.

12. Write the next paragraph of the review. Ethical and Legal Issues. Keep it short It should include the subjects: Informed consent challenges in prepubertal boys and Regulation and potential misuse of preserved samples.

13. Make it shorter, in one paragraph, and avoid making a conclusion - we have not finished our manuscript yet.

14. Give me 5 references for this last section

15. Write the last section of our review: The conclusion (80 words).

16. Suggest 5 keywords for this article, one of them should be ChatGPT

17. Suggest 5 different options of title for this review

**Supplementary Table 1. Verification of statements generated by ChatGPT.**

|  | **Article section** | **Sentences generated by ChatGTP** | **References** |
| --- | --- | --- | --- |
| 1 | Introduction | “Fertility preservation represents a critical concern within the broader framework of reproductive health, especially for those facing potential fertility risks.” | [1, 2] |
| 2 |  | “… advances in medicine continue to improve survival rates for conditions like cancer…” | [3, 4] |
| 3 |  | the focus is increasingly shifting towards enhancing the quality of life post-recovery, of which preserving the ability to have biological children plays a significant part. | [5, 6] |
| 4 |  | In this context, the necessity for effective fertility preservation strategies for men and prepubertal boys cannot be overstated. The latter group particularly presents unique challenges as they have not yet undergone the process of spermatogenesis, limiting the application of techniques readily available for adults. | [7, 8] |
| 5 | Overview of Spermatogenesis | This transformation unfolds within the seminiferous tubules of the testes, transitioning spermatogonial stem cells into mature spermatozoa. | [9, 10] |
| 6 |  | The initial proliferative phase involves spermatogonial stem cells undergoing self-renewing mitotic divisions and sequential differentiation, transitioning from type A to type B spermatogonia, and further into primary spermatocytes. | [9, 10] |
| 7 |  | In the subsequent meiotic phase, each primary spermatocyte undergoes two sequential meiotic divisions. | [9, 10] |
| 8* |  | Meiosis I results in a reduction of the diploid number of chromosomes to haploid, while Meiosis II segregates the sister chromatids into separate cells, generating four haploid secondary spermatocytes. | [9, 10] |
| 9 |  | Finally, the spermiogenic phase is marked by round spermatids undergoing critical morphological transformations to form mature spermatozoa. These changes encompass acrosome and flagellum development, nuclear condensation, and cytoplasmic shedding. | [9, 10] |
| 10* |  | Each stage of spermatogenesis, being sensitive to different factors, demands precision in fertility preservation strategies. |  |
| 11 | Fertility Preservation Methods for Adult Men | “A crucial aspect of male fertility preservation is sperm cryopreservation … The process begins with the collection of semen samples, the standard method being ejaculation.” | [11, 12] |
| 12* |  | “ In instances where conventional ejaculation is not viable, such as cases of retrograde ejaculation or ejaculatory duct obstruction, alternative approaches like electroejaculation or surgical sperm retrieval techniques like testicular sperm extraction (TESE) or microsurgical epididymal sperm aspiration (MESA) may be employed…” | [12, 13] |
| 13* |  | “Following collection, the semen sample undergoes a detailed analysis for key parameters like sperm count, motility, and morphology, determining the viability of freezing” |  |
| 14 |  | “The cryopreservation procedure employs controlled rate freezing, which initially involves slow cooling, followed by rapid plunging to the final storage temperature of -196 degrees Celsius in liquid nitrogen” | [14, 15] |
| 15 |  | “The cryopreservation procedure employs controlled rate freezing, which initially involves slow cooling, followed by rapid plunging to the final storage temperature of -196 degrees Celsius in liquid nitrogen, ensuring long-term sperm viability.” | [16, 17] |
| 16 |  | “Despite variability in survival rates due to pre-freezing sample quality and the specific freezing-thawing protocol used, the successful employment of cryopreserved sperm in assisted reproductive technologies (ART) confirms its effectiveness. This procedure thereby presents a reliable method for preserving fertility in adult men, providing a path towards future biological parenthood.” | [18, 19] |
| 17 | Fertility Preservation for Prepubertal Boys | “…prepubertal boys present unique challenges. Principally, these pertain to their sexual immaturity and the associated absence of mature sperm.” | [8, 20] |
| 18 |  | “The leading methods considered for fertility preservation in this demographic is testicular tissue cryopreservation.” | [21, 22] |
| 19 |  | “ This procedure involves the surgical removal of a small portion of testicular tissue, which harbors spermatogonial stem cells (SSCs), and its subsequent freezing.” | [21, 22] |
| 20 |  | These SSCs hold potential to initiate spermatogenesis in the future, when mn reimplanted or matured in vitro | [21, 22] |
| 21 |  | “These (promising options for utilizing cryopreserved testicular tissue) include autologous transplantation, heterologous transplantation, and in vitro maturation (IVM) of testicular tissue or isolated SSCs.” | [22] |
| 22 |  | “ However, it is essential to underscore that these potential methods, although promising, are still primarily within the research domain.” | [21, 22] |
| 23 |  | “Several critical issues, including the risk of reintroducing malignancy…” | [23, 24] |
| 24 | Ethical and Legal Issues | Navigating the ethical and legal landscape of fertility preservation, especially in prepubertal boys, poses unique challenges | [25, 26] |
| 25 |  | Informed consent is a complex issue, as the implications, potential benefits, and risks of an experimental technique like testicular tissue cryopreservation must be thoroughly understood by parents or guardians. | [27, 28] |
| 26 |  | Moreover, strict regulations are required to guard against potential misuse of cryopreserved samples | [29] |
| 27 |  | These include unambiguous rules regarding the authorization of sample use/disposal of unusude sample ensuring the protection of individual rights and legal integrity. | [27, 29] |

*statements found to be incorrect or partially inaccurate

References:

1. Taylor, J.F. and M.A. Ott, *Fertility preservation after a cancer diagnosis: a systematic review of Adolescents', Parents', and providers’ perspectives, experiences, and preferences.* Journal of pediatric and adolescent gynecology, 2016. **29**(6): p. 585-598.

2. Martinez, F., *Update on fertility preservation from the Barcelona International Society for Fertility Preservation-ESHRE-ASRM 2015 expert meeting: indications, results and future perspectives.* Hum Reprod, 2017. **32**(9): p. 1802-1811.

3. Miller, K.D., et al., *Cancer treatment and survivorship statistics, 2016.* (1542-4863 (Electronic)).

4. *Fertility preservation and reproduction in patients facing gonadotoxic therapies: an Ethics Committee opinion.* Fertil Steril, 2018. **110**(3): p. 380-386.

5. Chiba, K. and M. Fujisawa, *Fertility preservation in men with cancer.* Reprod Med Biol, 2014. **13**(4): p. 177-184.

6. Huleihel, M. and E. Lunenfeld, *Approaches and Technologies in Male Fertility Preservation.* Int J Mol Sci, 2020. **21**(15).

7. Wyns, C., et al., *Management of fertility preservation in prepubertal patients: 5 years’ experience at the Catholic University of Louvain.* Human Reproduction, 2011. **26**(4): p. 737-747.

8. Tran, K.T.D., et al., *Male fertility preservation and restoration strategies for patients undergoing gonadotoxic therapies†.* Biol Reprod, 2022. **107**(2): p. 382-405.

9. SF, G., *Developmental Biology*. Spermatogenesis, ed. t. edition. Sinauer Associates.

10. Neto, F.T., et al., *Spermatogenesis in humans and its affecting factors.* Semin Cell Dev Biol, 2016. **59**(1096-3634 (Electronic)): p. 10-26.

11. Grin, L., E. Girsh, and A. Harlev, *Male fertility preservation-Methods, indications and challenges.* Andrologia, 2021. **53**(2): p. e13635.

12. Agarwal, A., C. Ong, and D. Durairajanayagam, *Contemporary and future insights into fertility preservation in male cancer patients.* Transl Androl Urol, 2014. **3**(1): p. 27-40.

13. Tournaye, H., C. Krausz, and R.D. Oates, *Concepts in diagnosis and therapy for male reproductive impairment.* Lancet Diabetes Endocrinol, 2017. **5**(7): p. 554-564.

14. Sharma, R., et al., *Effect of sperm storage and selection techniques on sperm parameters.* Syst Biol Reprod Med, 2015. **61**(1): p. 1-12.

15. Hezavehei, M., et al., *Sperm cryopreservation: A review on current molecular cryobiology and advanced approaches.* Reprod Biomed Online, 2018. **37**(3): p. 327-339.

16. Schulz, M., et al., *Human sperm vitrification: A scientific report.* Andrology, 2020. **8**(6): p. 1642-1650.

17. Ozimic, S., H. Ban-Frangez, and M. Stimpfel, *Sperm Cryopreservation Today: Approaches, Efficiency, and Pitfalls.* Current Issues in Molecular Biology, 2023. **45**(6): p. 4716-4734.

18. Anger, J.T., B.R. Gilbert, and M. Goldstein, *Cryopreservation of sperm: indications, methods and results.* J Urol, 2003. **170**(4 Pt 1): p. 1079-84.

19. Tao, Y., et al., *Human sperm vitrification: the state of the art.* Reproductive Biology and Endocrinology, 2020. **18**(1): p. 17.

20. Chen, L., Z. Dong, and X. Chen, *Fertility preservation in pediatric healthcare: a review.* Front Endocrinol (Lausanne), 2023. **14**: p. 1147898.

21. Tran, K.T.D., et al., *Male fertility preservation and restoration strategies for patients undergoing gonadotoxic therapiesdagger.* Biol Reprod, 2022. **107**(2): p. 382-405.

22. Chen, L., Z. Dong, and X. Chen, *Fertility preservation in pediatric healthcare: a review.* Front Endocrinol (Lausanne), 2023. **14**(1664-2392 (Print)): p. 1147898.

23. Ahler, A., et al., *Metastatic neuroblastoma in fertility preservation biopsy of clinically normal testis: a case report.* Reprod Biomed Online, 2023. **47**(2): p. 103232.

24. Nguyen, H.T.K., et al., *Testicular involvement of acute lymphoblastic leukemia in children and adolescents: Diagnosis, biology, and management.* Cancer, 2021. **127**(17): p. 3067-3081.

25. Goossens, E., et al., *Fertility preservation in boys: recent developments and new insights (†).* Hum Reprod Open, 2020. **2020**(3): p. hoaa016.

26. Picton, H.M., et al., *A European perspective on testicular tissue cryopreservation for fertility preservation in prepubertal and adolescent boys.* Hum Reprod, 2015. **30**(11): p. 2463-75.

27. Petropanagos, A., *Testicular Tissue Cryopreservation and Ethical Considerations: A Scoping Review.* Journal of Bioethical Inquiry, 2017. **14**(2): p. 217-228.

28. Goossens, E., et al., *Fertility preservation in boys: recent developments and new insights (dagger).* Hum Reprod Open, 2020. **2020**(3): p. hoaa016.

29. Rogers, A., *Restricting access to reproductive uses of minors’ stored gonadal tissue.* The American Journal of Bioethics, 2013. **13**(3): p. 41-42.
